# Engaging with stakeholders in a research programme to promote implementation of pulmonary rehabilitation in Bangladesh: Challenges and opportunities

**DOI:** 10.7189/jogh.10.020384

**Published:** 2020-12

**Authors:** GM Monsur Habib, Md. Nazim Uzzaman, Poonam Malik, Roberto Rabinovich, Aftab Uddin, SM Rowshan Alam, Sian Williams, Hilary Pinnock

**Affiliations:** 1 Bangladesh Primary Care Respiratory Society (BPCRS), Khulna, Bangladesh; 2 NIHR Global Health Research Unit on Respiratory Health (RESPIRE), Usher Institute, University of Edinburgh, Edinburgh, UK; 3 ELEGI/Colt laboratory, Centre for Inflammation Research, QMRI, University of Edinburgh, Edinburgh, UK; 4 International Centre for Diarrhoeal Disease Research, Bangladesh (icddr,b), Dhaka, Bangladesh; 5 Rangpur Medical College, Rangpur, Bangladesh; 6 International Primary Care Respiratory Group (IPCRG), Edinburgh, UK

The engagement of stakeholders can improve research prioritisation, implementation, and outcomes [1]. Stakeholders may be defined as the individuals and organisations having an interest or ‘stake’ in the outcome of a programme or project [2]. Stakeholder engagement is a process of building relationships through communication and shared decision-making at all stages of the research cycle [3]. Although the concept has been well established in high income countries for at least two decades, even there the optimal timing and methods remain uncertain due to inadequate evaluation [1]. In contrast, it is still a new concept in many low and middle income countries (LMICs) [4] including Bangladesh. An estimated 85% of global investment in health and biomedical research is wasted each year because of redundancy, failure to prioritise the needs of stakeholders, poorly designed research methods, and inadequate reporting of study results [5]. Engaging with relevant stakeholders early during the prioritisation, design and planning stages of research could help divert appropriate resources and funding to much needed but low prioritised areas of health care. Despite availability of frameworks for stakeholder engagement, little is known about ‘why’, ‘who’, ‘how’, and ‘when’ engagement strategies are being implemented in LMICs to influence outcomes and if/how they achieve success.

The National Institute for Health Research (NIHR) Global Health Research Unit on Respiratory Health (RESPIRE) at The University of Edinburgh was established to reduce morbidity and mortality caused by respiratory diseases in South East Asia [6] and to build research capacity. A Stakeholder Engagement and Governance platform supports the four partner countries to mobilise national and international partners to improve research outcomes. In this viewpoint, we describe how one of the RESPIRE partners, the Bangladesh Primary Care Respiratory Society (BPCRS), organised stakeholder engagement in Bangladesh to support the implementation of pulmonary rehabilitation for patients with chronic respiratory diseases (CRDs). Pulmonary rehabilitation is a group-based programme of exercise, education, psychological and other support which improves health outcomes for people with chronic obstructive pulmonary disease (COPD) and other CRD [7]. We reflect on our experience of the process; the barriers, challenges and enablers that we encountered; and the value of the stakeholder engagement to our subsequent work with a view to providing guidance for others doing health research and quality improvement in health services.

## WHY DID WE ORGANISE STAKEHOLDER ENGAGEMENT?

CRDs such as COPD, asthma, post-tuberculosis lung disorders, bronchiectasis, occupational lung diseases and other often-unidentified chronic lung conditions affect an estimated 545 million people globally [8] and more than half of them live in LMICs [9]. Pulmonary rehabilitation is an integral component of the management of CRDs with proven effectiveness in reducing premature mortality, morbidity and improving functional capacity and health related quality of life [10]. However, typically there are no structured, evidence-based pulmonary rehabilitation (PR) facilities available in LMICs, especially in rural communities and sometimes services are underprovided even in high-income countries [11]. Building on a systematic review of the clinical effectiveness, components and delivery of pulmonary rehabilitation services in low-resource settings [12], and to complement on-going feasibility work, we planned a programme of stakeholder engagement in Bangladesh. A number of stakeholder engagement events were held with the aim of raising awareness of the benefits of pulmonary rehabilitation, and to understand the views of patients, public health officials, policymakers, politicians, religious leaders, and other stakeholders about the initiative, and to explore how they could influence adoption of the findings of our research.

## HOW DID WE IDENTIFY STAKEHOLDERS?

**Figure 1:**
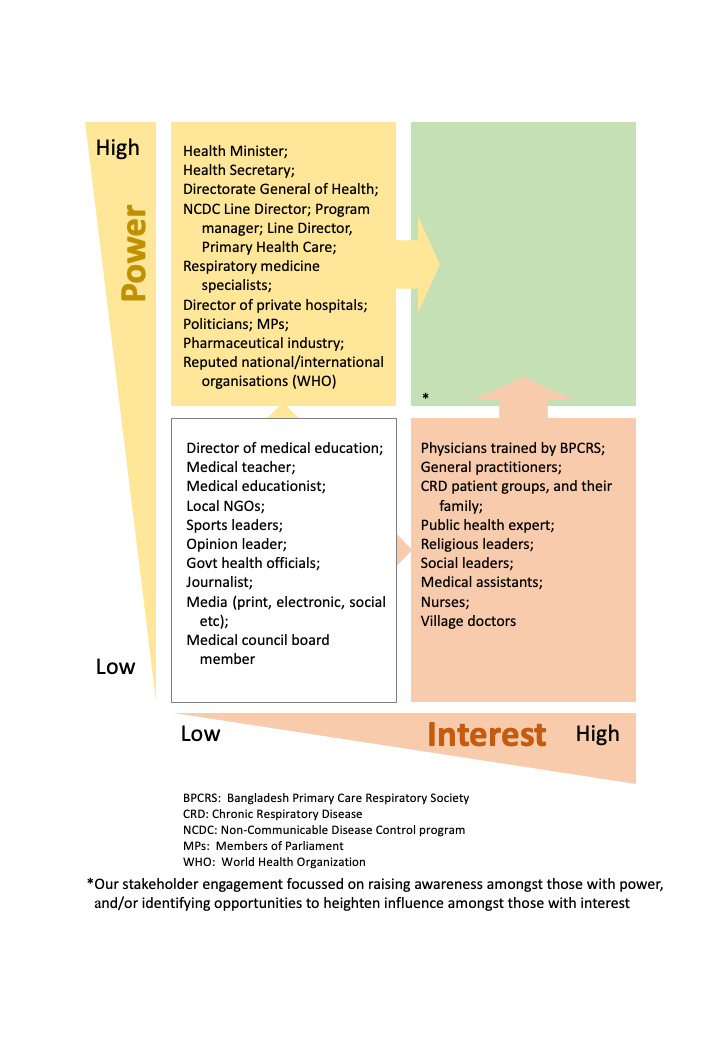
Mapping and indicating the migration of stakeholders according to their interest in pulmonary rehabilitation and their power to influence change in Bangladesh.

Early in the RESPIRE feasibility project implementing PR in Bangladesh, we used the ‘9Cs’ checklist (commissioners, customers, collaborators, contributors, channels, commentators, consumers, champions, competitors) recommended to ensure inclusion of all relevant stakeholders [13]. This facilitated ‘brainstorming’ to identify all the people and agencies likely to be involved in or affected by implementation of PR in the Bangladesh health care context. After that we used a ‘four-quadrant matrix’ to place the stakeholders according to their likely degree of interest or involvement, and our assessment of their power or influence over the conduct of the research (**Figure 1**). This ‘power-interest’ grid informed where to prioritise our engagement activities with efforts focussing on increasing interest amongst those with high power to block or enable change (migrating groups from the yellow to the green quadrant) and seeking to promote the influence of those with high interest (migrating from orange to green segment) The goal was to maximise the benefits from engagement activities by focusing our limited resources on those with (or potentially with) ‘high power’ and ‘high interest’ to help us improve the outcomes of our intervention.

**Figure:**
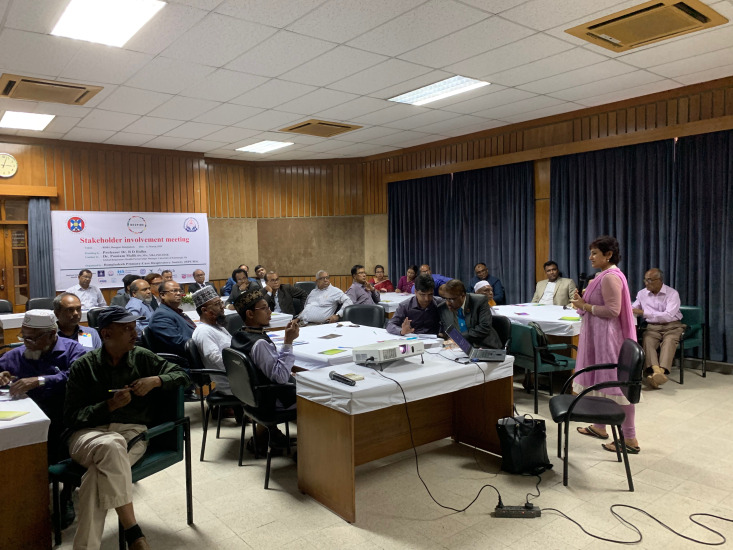
Photo: Engagement meeting in action (used with permission).

## HOW DID WE CONDUCT STAKEHOLDER ENGAGEMENT?

Having prioritised our actions, we planned a range of activities, reflecting on what gave us most traction with our key stakeholders. We conducted a broad range of activities promoting respiratory health in general and specifically advocating pulmonary rehabilitation at educational and clinical meetings, professional development opportunities, awareness-raising programmes, smoking cessation events, school-based programmes, community meetings, media coverage, and rallies over a considerable period of time. The explicit aim of these diverse stakeholder engagement activities, events and meetings was to raise awareness of the potential of pulmonary rehabilitation to reduce the burden of CRD in Bangladesh, especially amongst those with power to influence, and to optimise the potential of those with an interest to influence change.

In addition, we conducted six stakeholder engagement meetings in the only three community-based clinics currently offering PR in Bangladesh. One multi-professional group participated in Rangpur; a patient group and a multi-professional group in Khulna, and three groups in Dhaka (for policy makers and public health officials; multi-professional groups; and primary care physicians). As the capital city of Bangladesh, Dhaka was a convenient venue for involving policy makers. We sent an invitation by e-mail to targeted stakeholders, explaining our plans and followed this up by telephone regarding potential attendance at the stakeholder engagement meeting.

In each meeting, we facilitated discussion with the stakeholders on the following questions:

1. What are the challenges and barriers you think we might face in implementing pulmonary rehabilitation services in Bangladesh?

2. How do you think in your current role/position you can help us to enable implementation of pulmonary rehabilitation services in Bangladesh?

We also asked individual stakeholders what they thought was necessary to engage people in improving respiratory health in Bangladesh and to raise interest levels in implementation of PR services.

## WHAT DID WE FIND?

The most frequently identified challenges in implementing PR services were lack of research evidence on clinical effectiveness in Bangladesh, poor health literacy amongst patients, economic and cultural barriers, and lack of knowledge among health professionals. Stakeholders identified the need to train health care professionals, include pulmonary rehabilitation in the undergraduate medical curriculum, involve political and religious leaders who could exert influence, arrange a one-stop accessible service, and specifically to train therapists able to deliver pulmonary rehabilitation.

The most frequent responses to how we could enhance interest were to conduct awareness-raising programmes, increase media coverage, and address misconceptions about physical activity among older people. Regarding engagement, participation and removing barriers, the most common suggestions were developing patient groups, involving media, attracting the attention of the government policy makers and national parliament.

PR is not well known in LMICs, so we adapted a message from the International Primary Care Respiratory Group (IPCRG), “*Do you want to breathe and live better, feel good, and do more? – pulmonary rehabilitation will help you*” [14], which we hoped would begin to raise public awareness and generate interest amongst stakeholders. The challenge then was to convert the interest into action, and encourage those with influence to promote implementation of PR services in Bangladesh.

## CHALLENGES WE FACED

Before engaging with the study team, some stakeholders considered these meetings to be poor use of their time. However, many of them changed their views after attending a discussion and engagement session. Unfortunately, many potential stakeholders, such as tertiary care physicians identified as having high power and influence did not attend the meeting, despite responding positively to the telephone invitation. These are busy people with many calls on their time and influence, but in a hierarchical culture other factors may contribute, including that they were invited to be a participant (as opposed to a guest). The invitation came from a primary care group which may have reduced the priority that specialists attached to the event. Stakeholder engagement is an on-going process and further engagement efforts will be needed to overcome these challenges. Strategies that we are employing include taking every opportunity to attend and speak at their meetings and raising credibility by publishing our work in peer-reviewed journals as one of their preferred communication methods.

Although we planned multidisciplinary discussions, this was challenged by the cultural context, hierarchies and seating arrangement of venues. For example, higher ranking officials felt uncomfortable at being asked to participate in a group activity rather than being seated at the dias. Additionally, careful and sensitive facilitation was required to elicit responses to our questions rather than invitees giving pre-prepared answers based on their role and identity.

## PRACTICAL CONSIDERATIONS

Food and honoraria are important issues that need to be considered when organising stakeholder engagement meeting in LMICs like Bangladesh. Offering food is the cultural norm, and being paid an honorarium is an expectation both in public and private sectors; not doing so risks disappointment and discouraging participation in future events. Compensation for travel expenses or accommodation support (if someone had to travel long distance requiring overnight stay) is reasonable, but honoraria were not acceptable as the research programme was being funded as aid from UK public/ tax-payer’s funds. During COVID-19 such face-to-face activities will not be possible, so we will need to find new methods of stakeholder engagement.

## CONCLUSION

Implementation of sustainable pulmonary rehabilitation services in LMICs offers an exemplar for engagement with a broad range of stakeholders. With mentoring and support from RESPIRE colleagues, we used simple well-tested tools (the ‘9Cs’ checklist and the a four-quadrant matrix) and found them to be an effective approach to identifying stakeholders and prioritising our activities. We adopted a practical approach, employing strategies to raise awareness and interest especially amongst those with power and potential to influence. We had to tailor the engagement meetings to meet local social and cultural norms but believe that we succeeded in raising the profile of CRD and the role of pulmonary rehabilitation and in identifying strategies that we can take forward. We hope our experiences will help other researchers in LMICs to conduct successful stakeholder engagement to enhance effective implementation of research outcomes and interventions.
